# Monitoring Minimal Residual Disease in the Myeloproliferative Neoplasms: Current Applications and Emerging Approaches

**DOI:** 10.1155/2016/7241591

**Published:** 2016-10-20

**Authors:** Karl Haslam, Stephen E. Langabeer

**Affiliations:** Cancer Molecular Diagnostics, St. James's Hospital, Dublin 8, Ireland

## Abstract

The presence of acquired mutations within the* JAK2*,* CALR,* and* MPL* genes in the majority of patients with myeloproliferative neoplasms (MPN) affords the opportunity to utilise these mutations as markers of minimal residual disease (MRD). Reduction of the mutated allele burden has been reported in response to a number of therapeutic modalities including interferon, JAK inhibitors, and allogeneic stem cell transplantation; novel therapies in development will also require assessment of efficacy. Real-time quantitative PCR has been widely adopted for recurrent point mutations with assays demonstrating the specificity, sensitivity, and reproducibility required for clinical utility. More recently, approaches such as digital PCR have demonstrated comparable, if not improved, assay characteristics and are likely to play an increasing role in MRD monitoring. While next-generation sequencing is increasingly valuable as a tool for diagnosis of MPN, its role in the assessment of MRD requires further evaluation.

## 1. Introduction

The Philadelphia chromosome-negative myeloproliferative neoplasms (MPN) are clonal hematopoietic diseases characterised by bone marrow proliferation of one or more of the myeloid cell lineages with no marked alterations in cellular maturation. MPNs classically comprise the clinically and pathologically related polycythemia vera (PV), essential thrombocythemia (ET), and primary myelofibrosis (PMF). In PV and ET, the potential exists for the disease to transform to a myelofibrotic stage and, together with PMF, transform to acute myeloid leukemia. Identification of the* JAK2* V617F mutation, now over a decade ago, revolutionised the molecular diagnosis of MPN as this mutation is present in up to 95% of patients with PV and in approximately 50–60% of patients with ET and PMF. Subsequent identification of further disease initiating mutations, such as those in* JAK2* exon 12,* MPL* exon 10, and* CALR* exon 9, allows the implementation of molecular diagnostic algorithms that are able to identify a clonal marker of disease in the vast majority of classical MPN patients. Numerous other myeloid-associated mutations have been additionally detected in MPN patients whose acquisition appears to influence the phenotype and disease course [1]. Several laboratory approaches are available for the identification of these mutations at diagnosis with selection of methodology largely dependent on the clinical utility required [2].

The primary treatment goals in MPN are to avoid thrombosis and bleeding, treat MPN related symptoms, improve quality of life, and minimize risk of malignant transformation and/or post-ET/PV myelofibrosis. However, the presence of the* JAK2*,* CALR*, and* MPL* “driver” mutations allows them to be utilised for diagnosis, but quantitatively, they may serve as markers of minimal residual disease (MRD), an additional, valuable indicator of depth of response to therapeutic intervention. To date, the clinical validity of determining* JAK2*,* CALR*, and* MPL* MRD responses has been demonstrated with several modalities including interferon alpha, JAK 1/2 inhibitors, and allogeneic stem cell transplantation (ASCT). Novel targeted agents for MPN in development include specific JAK2 inhibitors, histone deacetylase inhibitors, hypomethylating agents, heat shock protein 90 inhibitors, PI3-AKT-mTOR inhibitors, and telomerase inhibitors, all of which, alone or in combination with established therapies, will require assessment of efficacy [3].

The presence of recurrent point mutations facilitates the use of allele-specific quantitative PCR (qPCR) which has become widely adopted. Emerging approaches and platforms that are becoming increasingly implemented in the diagnostic setting with potential application for MRD monitoring include digital PCR (dPCR) and next-generation sequencing (NGS). This review will provide a brief overview of those methods already integrated into practice and consider those in development in regard to their clinical applicability to monitor MRD with respect to the three main types of MPN driver mutations.

## 2. Current Applications

Initial Sanger sequencing was superseded by pyro-sequencing, melt curve analysis, and allele-specific PCR for routine diagnostics because of its limited sensitivity and therefore inability to detect low mutation allele burdens. Remaining diagnostic methodologies vary in their sensitivity but should allow for the routine detection of the* JAK2* V617F or* MPL* exon 10 point mutations at an allele burden of approximately 1–3% [4, 5]. Assessment of MRD requires quantitation over a dynamic range of at least three logs, and taking heed of the acceptance of* BCR-ABL1* monitoring into the routine clinical management of chronic myeloid leukemia, a variety of allele-specific qPCR approaches have been widely adopted. Comparative studies have demonstrated the superior sensitivity of qPCR especially after calibration to common standards [6, 7]. Several primer/probe combinations have been used to quantitate the* JAK2* V617F allele burden by qPCR [8, 9] but assays can vary markedly in their performance. In order to address this issue and establish a consistent approach, a network of centres systematically evaluated several qPCR assays and was able to recommend the most consistently performing assay, suitable for assessing response in clinical trials, predicting outcome and guiding management of patients post-ASCT [10]. Mutations of* MPL* exon 10 are present in approximately 5% of ET and <10% of PMF patients. This low frequency, compared to the* JAK2* V617F and* CALR* exon 9 mutations, may explain that while several, sensitive qPCR-based techniques have been described for detection of the* MPL* exon 10 mutations at diagnosis [11–13], there remains a lack of information regarding their utility as markers of MRD in these MPN. Insertion/deletion (indel) mutations of* CALR* exon 9 are the most recently discovered MPN driver mutations. While semiquantitative, diagnostic fragment length analysis (FLA) has been extensively embraced, qPCR methods have been hampered by the variety of indels observed. However, recent studies focusing on the common type 1 deletion and type 2 insertion mutations that account for approximately 80% of all indels have exhibited improved specificity and sensitivity necessary for MRD purposes [14].

### 2.1. *JAK2* Mutations

As the* JAK2* V617F is the most frequent mutation in MPN, considerable effort has been afforded in addressing the clinical usefulness of using this mutation as an MRD marker in several treatment modalities. Hydroxyurea (HU) is one of the most widely used cytoreductive agents used in the treatment of PV and ET with initial reports suggesting that HU was able to suppress* JAK2* V617F levels by >30% in approximately half of patients, a phenomenon observed early in disease course [15, 16]. Where partial molecular responses are recorded, they appear more likely in those patients with a high* JAK2* V617F allele burden before treatment [17]. Subsequent studies with considerably longer follow-up, however, imply that HU does not appreciably reduce the* JAK2* V617F allele burden which either remains stable or only exhibits minor fluctuations over prolonged treatment periods [18–20].

Interferon alpha (IFN-*α*) is able to induce hematological responses in MPN patients but its use has been constrained by the side effects of the relatively large doses employed. Nonetheless, sustained molecular responses are observed in the majority of PV and ET patients who remain on treatment with complete elimination of the detectable* JAK2* V617F clone in selected cases [21–23]. Rare cases have been described in which this complete molecular response is remarkably maintained for a number of years after cessation of IFN-*α* [24, 25]. More recent clinical trial data point to less significant molecular responses in those patients with higher pretreatment allele burdens and in those with a higher incidence of disease modifying mutations [26]. Busulfan therapy for PV has lessened amid concerns of its leukemogenic potential; however, even in a small cohort of PV patients refractory to multiple therapies, busulfan induced considerable* JAK2* V617F responses, including complete molecular remission, in the majority of patients [27].

The discovery that the JAK-STAT signalling pathway, required for normal hematopoietic signalling, is disrupted by mutations in the majority of MPN patients makes this pathway an obvious target for therapeutic intervention. Several inhibitors of both wild-type and mutant JAK1 and JAK2 molecules have been developed. Despite substantial improvements in reducing spleen size, improving constitutional symptoms, and prolonging survival in PMF patients, JAK inhibitors fail to significantly reduce the disease bulk as evidenced by a reduction of the* JAK2* V617F load in those mutation positive patients [28, 29]. However, with longer follow-up of these landmark clinical trials, >10% of patients still on drug achieved either a partial or a complete molecular response with greater reductions in patients with shorter disease duration [30]. While the overall reduction in* JAK2* V617F allele burden remains modest in most patients treated with JAK 1/2 inhibitors, there is increasing evidence of slow but complete molecular responses in those patients on long term therapy justifying continued MRD assessment [31, 32].

Despite the introduction of JAK inhibitors for the treatment of PMF and HU-resistant PV, ASCT remains the only potentially curative option for those patients with advanced disease. Improvements in candidate patient selection and stratification, timing of transplantation, and conditioning regimens have significantly reduced the transplant related morbidity and increased the overall survival for patients undergoing this procedure [33]. Post-ASCT monitoring utilising additional patient-specific markers in addition to donor-recipient chimerism status is likely to provide a more beneficial, individualized approach. However, as relapse is a major cause of treatment failure after ASCT with salvage options limited and subsequent outcome relatively poor, identification of those patients at high-risk of relapse would be highly desirable, potentially enabling therapeutic intervention before overt relapse. Establishment of sensitive qPCR assays has retrospectively demonstrated the utility of this approach in detecting both disease clearance and persistence [10, 34–37]. Prospective monitoring allows consideration of preemptive adoptive immunotherapy with donor lymphocyte infusion in those cases with persistent or rising MRD [38, 39]. MRD assessment in the period immediately after ASCT should be considerably more frequent than in other modalities, perhaps on a weekly basis; one study has suggested that the level of MRD below or above 1.0% at one month after ASCT is highly predictive of outcome and relapse risk, respectively [40].

Mutations of* JAK2* exon 12 are present in the small percentage of PV patients who are* JAK2* V617F-negative. These* JAK2* exon 12 mutations, which can be substitutions, deletions, insertions, and duplications, are usually detected with a low allelic burden of <10% making quantitative MRD assessment problematic [41]. Individual qPCR assays for some common exon 12 mutations have been developed and demonstrate stability over time of both hetero-and homozygous allele burdens [42].

### 2.2. *MPL* Exon 10 Mutations

The most common* MPL* mutations are W515L and W515K with several other point mutations noted in codon W515 and elsewhere within exon 10 [43]. Although amenable to allele-specific qPCR approaches, their low frequency in ET and PMF, compared to the* JAK2* V617F and* CALR* exon 9 mutations, has limited their use as MRD markers. In the post-ASCT setting, qPCR of both* MPL* W515L and W515A mutations in individual patients has demonstrated clearance of MRD in PMF patients [44, 45]. Lowering of* MPL* W515L/K allele burdens has also been demonstrated in ET patients treated with a telomerase inhibitor [46].

### 2.3. *CALR* Exon 9 Mutations

The diagnostic method of choice for* CALR* mutations in ET and PMF must have the ability to detect the plethora of indels thus far reported. Several approaches have been utilised with their sensitivities compared [47]; fragment length analysis (FLA) followed by capillary electrophoresis has been widely adopted and displays sensitivity compatible for MRD monitoring (approximately 1%) but remains semiquantitative [48–50]. Analysis of* CALR* mutation by FLA has been retrospectively applied to patients with PMF after ASCT and has mirrored donor chimerism status in those relapsing patients demonstrating proof of principle [51]. In a cohort of 31 ET patients with* CALR* mutations with considerable follow-up, interferon significantly decreased the* CALR* mutant allele burden with some patients achieving complete molecular remission; of further note, the presence of additional mutations was associated with a poorer molecular response [52]. In a retrospective analysis of a pivotal phase 3 study of PMF and post-ET PMF patients, a JAK 1/2 inhibitor demonstrated clinical effectiveness in* CALR*-mutated patients but the allelic ratio as performed by FLA was not significantly lowered at a median of 60 weeks of treatment; however a small number of patients displayed molecular responses with longer follow-up [53].

In order to improve sensitivity and given the variety of* CALR* indels reported, qPCR approaches have focused on the two major mutations of type 1 (52 bp deletion) and type 2 (5 bp insertion) that account for more than 80% of* CALR* mutations. Initial attempts for qPCR of these common* CALR* mutations resulted in limited sensitivities of 1-2%, comparable to other molecular screening approaches [54]. Development of alternative qPCR assays has managed to further improve the sensitivity for both type 1 and type 2* CALR* mutations [14].

## 3. Emerging Approaches

### 3.1. Digital PCR

An emerging approach for the detection and quantitation of MPN-associated mutations is digital PCR (dPCR) which can achieve quantitation of the target allele without the requirement for standard curve construction or comparison to a reference gene. Partition of the template DNA into multiple PCR reactions is achieved by either droplet formation or nanofluidics, resulting in improved sensitivity and accuracy with minimal requirements for validation and standardization [55]. Comparisons of dPCR and qPCR have revealed strong correlations in the quantitation of the* JAK2* V617F, even at low allele burdens of 0.1%, indicating clinical utility for MRD monitoring [56–58]; however further studies are required to establish this capability.

The accurate determination of mutant* CALR* allele burdens by dPCR has recently been applied to PMF patients in the post-ASCT setting with considerable improvements observed in the detection and quantitation of type 1 and type 2* CALR* mutants. As compared to FLA or qPCR, dPCR was capable of detecting delayed clearance of MRD after ASCT and earlier detection of increasing MRD levels prior to clinical relapse [59, 60]. Sequential dPCR evaluations in a limited number MPN patients treated with interferon have shown reductions in the* CALR* mutant allele burden and, in one case, disappearance of the mutant clone at the time of transformation to acute myeloid leukemia [61].

### 3.2. Next-Generation Sequencing

The capacity of next-generation sequencing (NGS) technologies, platforms, and accompanying bioinformatics pipelines to simultaneously sequence multiple genes and identify mutations with high specificity and comparable sensitivity lends itself to MPN molecular diagnostics in which a considerable degree of genomic complexity occurs. Targeted exon sequencing allows the identification of several mutations that not only demonstrate clonality but also have prognostic and therapeutic significance [62, 63]. While acquired mutations at diagnosis by NGS in myelodysplastic/myeloproliferative neoplasms may be used as subsequent markers of MRD after ASCT [64], the inadequate dynamic range of detection of NGS approaches has limited their assignment to routine MRD purposes. One encouraging study has demonstrated that increasing the number of NGS reads allows improved assessment of* JAK2* V617F MRD levels and demonstrated appreciable correlation with a qPCR approach [65].

## 4. Further Considerations

Implementation of which MRD approach to adopt requires consideration of which cohort of patients to screen and technical aspects of the selected assays as discussed above. In addition harmonization of MRD data requires efficient handling and standardization of how results are to be reported [66] and also what options are available for both continued compliance and prospective proficiency evaluation. In order to audit this proficiency, internationally recognized standards are required. While an increased frequency of MRD assessment is required after ASCT the optimal testing frequency requires establishing for each therapeutic modality. Evaluation of standardized molecular responses is likely to be incorporated in clinical trials of novel targeted agents and potential drug combinations for the treatment of MPN, which is not reflected in current guidelines [67].

The identification of multiple gene defects and deregulated signalling pathways in MPN has, in turn, propagated expansion of clinical research into specific inhibitors targeting these aberrations. The addition of MRD monitoring is likely to supplement these interventions in providing an individualized pathway of patient management.
